# Impact of Concomitant Metamizole Treatment on the Exposure of Mould‐Active Triazoles

**DOI:** 10.1111/myc.70173

**Published:** 2026-05-14

**Authors:** Dorian Vanneste, Ine De Booser, Kristina Nadrah, Matej Somrak, Matthias Gijsen, Aleš Matos, Katja Kalan, Bart Morlion, Steffen Rex, Saba Battelino, Katrien Lagrou, Johan Maertens, Isabel Spriet

**Affiliations:** ^1^ Department of Pharmaceutical and Pharmacological Sciences, Clinical Pharmacology and Pharmacotherapy KU Leuven Leuven Belgium; ^2^ Department of Infectious Diseases University Medical Center Ljubljana Slovenia; ^3^ Institute of Clinical Chemistry and Biochemistry University Medical Center Ljubljana Slovenia; ^4^ Pharmacy Department University Hospitals Leuven Leuven Belgium; ^5^ Grošelj Matos Otorinolaringologija Ljubljana Slovenia; ^6^ Pharmacy Department University Medical Centre Ljubljana Slovenia; ^7^ Department of Anaesthesiology UZ Leuven Leuven Belgium; ^8^ Department of Cardiovascular Sciences KU Leuven Leuven Belgium; ^9^ Department of Otorhinolaryngology and Cervicofacial Surgery University Medical Center Ljubljana Slovenia; ^10^ Faculty of Medicine, Department of Otorhinolaryngology University of Ljubljana Ljubljana Slovenia; ^11^ Department of Microbiology Immunology and Transplantation, KU Leuven Leuven Belgium; ^12^ Department of Laboratory Medicine and National Reference Centre for Mycosis University Hospitals Leuven Leuven Belgium; ^13^ Haematology Unit, UZ Leuven Leuven Belgium

**Keywords:** drug interactions, metamizole, mould‐active triazoles

## Abstract

**Aim:**

Metamizole is an analgesic drug with moderate cytochrome P450 (CYP) inductive properties. The antifungal triazoles are metabolized by several CYP enzymes, but the interaction with metamizole remains poorly described. We investigated the influence of metamizole on the exposure of voriconazole, isavuconazole, and posaconazole.

**Methods:**

Patients from UZ Leuven (Belgium) and Ljubljana University Medical Centre (Slovenia) receiving voriconazole, isavuconazole, or posaconazole concomitantly with metamizole were included in the study. Routine therapeutic drug monitoring (TDM) measurements collected between January 2019 and December 2024 were retrieved retrospectively. TDM concentrations outside of concomitant therapy were collected as controls. The influence of metamizole and other clinically relevant covariates was analysed using generalized estimating equations (GEE).

**Results:**

A total of 126 distinct treatments with a triazole from 115 patients, accounting for 392 measurements, were included in the study. GEE analysis revealed a significant negative association between the 7‐day cumulative metamizole dose and lower voriconazole and posaconazole concentrations. Additionally, C‐reactive protein had a positive association with voriconazole concentrations. Only ICU admission and patient characteristics, that is, sex and weight, had a significant influence on isavuconazole concentrations.

**Conclusion:**

Concomitant therapy with metamizole led to lower voriconazole and posaconazole concentrations, presumably through induction of CYP enzymes and possibly UDP‐glucuronyltransferase. We recommend avoiding concomitant use of metamizole with the antifungal triazoles to prevent underexposure and treatment failure or frequent TDM if the combination cannot be avoided. Further studies are needed to confirm our findings and investigate the influence of metamizole on other triazoles.

## 1 Introduction

Metamizole, also known as dipyrone, is a pyrazolone derivate with analgesic, antipyretic, and spasmolytic properties. Despite its strong analgesic effect and good gastrointestinal tolerability, the use of metamizole has been limited in some Western countries because of the unpredictable dose‐independent risk of severe agranulocytosis [1, 2]. However, during the COVID pandemic, fears emerged that non‐steroidal anti‐inflammatory drugs and paracetamol could be associated with negative clinical outcomes [3] which led to a renewed interest in metamizole as an alternative analgesic and antipyretic option. Currently, metamizole is frequently used as an analgesic for moderate to severe post‐operative pain and fever, especially in those for whom other analgesics are contraindicated [1].

Metamizole and its metabolites, 4‐methylaminoantipyrine and aminoantipyrine, are known inducers of cytochrome P450 (CYP) enzymes [1]. In vitro hepatocyte studies have confirmed an increase in expression of CYP2B6, CYP2C9, CYP2C19, and CYP3A4 after exposure to metamizole [4, 5]. The induction is mediated through the constitutive androstane receptor (CAR) [5]. CYP3A4 activity is significantly higher after 3 days of metamizole therapy and reaches peak induction after 6 days. This effect can last up to 5 days after cessation of metamizole treatment [6]. Induction of CYP enzymes by metamizole has the potential to cause significant drug–drug interactions (DDI). A recent review summarized the pharmacokinetic and pharmacodynamic interactions of metamizole with various compounds. The review clearly highlighted the clinical relevance of broad CYP induction by metamizole [7]. However, little is known about the impact of metamizole on the antifungal triazoles.

The mould‐active triazoles are a class of antifungal drugs used for prophylaxis and treatment of invasive mould infections, such as invasive aspergillosis and mucormycosis. These infections are associated with high morbidity and mortality; thus, early adequate treatment is essential. Because of a wide intra‐ and interpatient variability, therapeutic drug monitoring is carried out in daily clinical practice. Triazoles are primarily cleared hepatically by CYP enzymes such as CYP3A4, CYP2C9, and CYP2C19 or by UDP‐glucuronyltransferase (UGT). In addition to being CYP substrates, most triazoles also inhibit CYP3A4 themselves. Interactions with both CYP3A4 substrates or inducers have been described [8, 9, 10]. However, to date, only two articles describe the interaction between metamizole and voriconazole [11, 12]. Reports on the impact of metamizole on the exposure of other mould‐active triazoles are currently unavailable.

This study aims to explore and describe a potential interaction between metamizole and voriconazole, posaconazole, and isavuconazole.

## 2 Methodology

### 2.1 Study Design

The study is a retrospective exploratory study in two tertiary care centers, UZ Leuven (Belgium) and the Ljubljana University Medical Centre (Slovenia). The study was approved by the ethics committee of UZ Leuven (S65215) and the National Medical Ethics Committee of the Republic of Slovenia (0120‐12/2025‐2711‐3). Individual informed consent was not required for this study, in compliance with institutional and national ethical standards. All hospitalized patients between a period of January 2019 and December 2024 were screened for eligibility. Patients who were concomitantly treated with metamizole and either voriconazole, isavuconazole, or posaconazole, and with at least one routine trough concentration (Cmin) sample collected as part of routine therapeutic drug monitoring (TDM) could be included in the study. Concomitant treatment was defined as receiving any dose of metamizole in a period of 7 days before the day of TDM. A 7‐day threshold was selected to capture both the peak induction phase and the residual effects of CYP enzyme induction [6]. Patients under 2 years of age were not included in the study, as CYP3A4 enzyme activity is not expected to be fully mature before this age [13].

The following data were collected from eligible patients: demographic data at time of admission; dosing and timing of metamizole and the relevant triazole; triazole Cmin and date; the 7‐day cumulative metamizole dose prior to TDM. Patient data was obtained from the electronic healthcare systems of both hospitals. Separate triazole treatment episodes within the same patient were treated as unique events, considering that baseline characteristics may vary across different treatment episodes within the same individual.

Triazole Cmin measured in included patients in the absence of concomitant therapy were collected as within‐patient controls. Therapeutic ranges for the azoles were defined as Cmin between 2–5.5 mg/L for voriconazole, 2–5 mg/L for isavuconazole, and 1–3.75 mg/L for posaconazole [8].

### 2.2 Statistical Analysis

All statistical analyses were performed using R software (R version 3.5.1 or higher, R Core Team, Vienna, Austria). A multivariable analysis for voriconazole and isavuconazole, and univariate analyses for posaconazole were performed to assess the impact of clinically relevant (age, sex, body weight, ICU admission, C‐reactive protein (CRP) levels on day of TDM) and pharmacologically relevant covariates (triazole dose, administration route of the triazole, and the 7‐day cumulative metamizole dose prior to TDM) on the triazole Cmin. The uni‐ and multivariable analyses were performed using the “geepack”‐package for generalized estimating equations (GEE) [14] using an exchangeable correlation structure. The estimated effect was reported with its standard error and *p*‐value, which was considered statistically significant if less than 0.05.

## 3 Results

A total of 115 patients were included in the study which accounted for 126 distinct triazole treatment episodes. The demographics describing the study population are shown in Table 1. Of the 392 routine Cmin, 200 were collected during concomitant therapy with metamizole (Concomitant), and 192 outside of concomitant metamizole therapy (Control).

**TABLE 1:** Baseline demographics of the study population.

Characteristics	Overall (*N* = 126)
Age (years)	62.5 [51.3–69.0]
Female	40 (31.7%)
Body weight (kg)	76.5 [63.4–88.8]
ICU admission	58 (46.0%)
Triazole therapy	
Voriconazole	76 (60.3%)
Isavuconazole	34 (27.0%)
Posaconazole	16 (12.7%)

*Note:* Categorical variables are presented as frequency (%). Continuous variables are presented as median [Q1‐Q3].

As shown in Table 2, the median CRP concentration was numerically higher in all three concomitant treatment groups. Concerning target attainment, the proportion of Cmin within therapeutic range was lower in the concomitant group for isavuconazole (54.7% vs. 74.3%) and posaconazole (63.2% vs. 81.8%). Similarly, azole Cmin was more frequently subtherapeutic in the concomitant group of isavuconazole (35.8% vs. 24.3%) and posaconazole (36.8% vs. 18.2%). These trends were not seen for voriconazole, which had a relatively low target attainment of 12.5% and 19.5% in the control and concomitant groups, respectively. The proportion of supratherapeutic voriconazole Cmin was lower in the concomitant group (47.7% vs. 53.2%). Median 7‐day cumulative metamizole dose was highest in the voriconazole group, followed by the posaconazole and isavuconazole groups. Patients with sub‐therapeutic posaconazole and voriconazole Cmin, had a numerically higher cumulative 7‐day metamizole dose compared to patients with therapeutic or supra‐therapeutic Cmin (Table S1).

**TABLE 2:** Routine TDM concentrations by triazole and associated characteristics.

Characteristics	Voriconazole		Isavuconazole		Posaconazole	
	Control (*n* = 111)	Concomitant (*n* = 128)	Control (*n* = 70)	Concomitant (*n* = 53)	Control (*n* = 11)	Concomitant (*n* = 19)
Measured triazole Cmin (mg/L)	2.90 [1.30–4.30]	3.00 [1.20–4.73]	2.45 [2.00–3.00]	2.20 [1.50–3.50]	1.30 [1.05–1.60]	1.30 [0.750–1.50]
24 h triazole dose (mg)	600 [400–800]	600 [400–728]	200 [200–300]	200 [200–200]	400 [300–400]	300 [300–300]
24 h weight‐adjusted triazole dose (mg/kg)	8.24 [7.14–10.6]	7.70 [6.16–8.95]	3.58 [2.62–4.40]	2.82 [2.36–4.17]	4.90 [3.94–5.49]	4.10 [3.71–4.29]
Administration routes of the triazole						
IV	74 (66.7%)	81 (63.3%)	53 (75.7%)	38 (71.7%)	5 (45.5%)	8 (42.1%)
IV and Oral	7 (6.3%)	6 (4.7%)	3 (4.3%)	1 (1.9%)	1 (9.1%)	5 (26.3%)
Oral	30 (27.0%)	41 (32.0%)	14 (20.0%)	14 (26.4%)	5 (45.5%)	6 (31.6%)
7‐day cumulative metamizole dose (mg)	0 [0–0]	2500 [1000–10,000]	0 [0–0]	1500 [500–3500]	0 [0–0]	2100 [750–3250]
CRP (mg/L)	44.4 [19.0–130]	63.7 [26.4–186]	43.2 [23.5–104]	137 [48.7–264]	30.6 [8.65–80.2]	84.9 [52.5–291]
Target attainment						
Sub‐therapeutic	38 (34.2%)	42 (32.8%)	17 (24.3%)	19 (35.8%)	2 (18.2%)	7 (36.8%)
Therapeutic	14 (12.6%)	25 (19.5%)	52 (74.3%)	29 (54.7%)	9 (81.8%)	12 (63.2%)
Supra‐therapeutic	59 (53.2%)	61 (47.7%)	1 (1.4%)	5 (9.4%)	0 (0%)	0 (0%)

*Note:* Continuous variables are presented as median [Q1‐Q3]. Categorical variables are presented as frequency (%). Therapeutic ranges for the azoles were defined as Cmin between 2–5.5 mg/L for voriconazole, 2–5 mg/L for isavuconazole, and 1–3.75 mg/L for posaconazole (8).

Abbreviations: Cmin, trough concentration; CRP, C‐reactive protein; IV, intravenous.

Multivariable analysis of voriconazole showed a significant impact of the patient's age (*p* = 0.029), CRP concentration (*p* < 0.001), and 7‐day cumulative metamizole dose (*p* = 0.007) on voriconazole Cmin. For isavuconazole, sex (*p* = 0.004), body weight (*p* < 0.001), type of ward (*p* = 0.021), and isavuconazole dose in the 24 h prior to TDM (*p* = 0.048) were found to be significantly associated. Considering the low sample size of posaconazole, only a univariate analysis was performed. This revealed a significant influence of posaconazole dose in the 24 h prior to TDM (*p* < 0.001) and 7‐day cumulative metamizole dose (*p* < 0.001). Full outputs of all the analyses are shown in Tables 3 and 4.

**TABLE 3:** Multivariable analysis of voriconazole and isavuconazole. Std. error = standard error. DM7 = median cumulative metamizole dose in a period of 7 days before the triazole concentration was measured.

	Voriconazole			Isavuconazole		
	Estimate	Std. error	*p*	Estimate	Std. error	*p*
Age	0.03	0.01	**0.029**	0.01	0.01	0.402
Female sex	−0.24	0.39	0.537	−0.70	0.24	**0.004**
Body weight (kg)	−1.05e‐03	0.02	0.956	−0.02	5.63e‐03	**< 0.001**
ICU admission	−0.02	0.50	0.970	−1.02	0.44	**0.021**
C‐reactive protein (mg/L)	0.01	3.03e‐03	**< 0.001**	1.91e‐04	1.52e‐03	0.900
24 h triazole dose (mg)	1.28e‐03	1.31e‐03	0.332	2.15e‐03	1.09e‐03	**0.048**
Administration route of the triazole Ref: Oral						
IV	0.41	0.45	0.362	0.14	0.53	0.799
Oral and IV	−0.13	0.52	0.802	−0.17	0.52	0.740
7‐day cumulative metamizole dose (mg)	−6.58e‐05	2.43e‐05	**0.007**	7.25e‐05	7.35e‐05	0.324

*Note:* Values in bold highlight *p*‐values < 0.05.

**TABLE 4:** Univariate analysis of Posaconazole.

Covariates	Estimate	Standard error	*p*
Age	−2.41e‐03	4.71e‐03	0.61
Female sex	0.22	0.28	0.42
Body weight (kg)	−4.84e‐03	3.43e‐03	0.16
ICU admission	−0.03	0.36	0.92
C‐reactive protein (mg/L)	6.96e‐04	8.86e‐04	0.43
24 h posaconazole dose (mg)	3.27e‐03	0.56e‐03	**< 0.001**
Administration route of the triazole Ref: Oral			
IV	−0.22	0.23	0.35
Oral and IV	−0.39	0.23	0.10
7‐day cumulative metamizole dose (mg)	−8.89e‐05	5.37e‐06	**< 0.001**

*Note:* Values in bold highlight *p*‐values < 0.05.

## 4 Discussion

We retrospectively investigated the influence of concomitant metamizole therapy on the exposure of mould‐active triazoles. Considering our sample size, we were able to perform multivariable analyses using GEE models for voriconazole and isavuconazole, and an univariable analysis for posaconazole. We found a significant negative association of the 7‐day cumulative metamizole dose with the trough concentrations of voriconazole and posaconazole. Such an association was not found for isavuconazole. To date, this represents the largest study exploring the influence of metamizole on the mould‐active triazoles.

In our study, concomitant treatment of mould‐active triazoles with metamizole was associated with a numerically higher proportion of subtherapeutic Cmin for isavuconazole and posaconazole. This effect has previously been shown as well for voriconazole by Horcada et al. [11] and Baan et al. [12]. Horcada et al. [11] presented two cases where metamizole was found to be the cause of reduced voriconazole concentrations. Baan et al. [12] compared voriconazole concentrations before, during, and after metamizole treatment in a small cohort of nine patients. They found that during metamizole treatment, voriconazole concentrations decreased by 70% compared to concentrations measured prior to concomitant therapy. This is in contrast with our findings as we did not notice a difference in voriconazole concentrations during concomitant therapy with metamizole. However, there were some notable differences with our study. First, the timeframe used to define concomitant therapy was 7 days in our study, compared to the 5‐day window in their study. Additionally, we did not require a minimum duration of metamizole exposure prior to inclusion. Second, the proportion of subtherapeutic concentrations in their control group was lower compared to ours (14% vs. 34%). This is probably attributable to our use of a higher threshold of 2 mg/L for the therapeutic range.

Multivariable analysis identified a statistically significant association between voriconazole Cmin and the 7‐day cumulative metamizole dose, age, and CRP concentration. A negative association was found with the 7‐day cumulative dose, that is, a higher 7‐day cumulative metamizole dose was associated with a lower voriconazole exposure. This result aligns with the findings from previous studies on metamizole‐associated CYP‐induction [12, 15]. Age and CRP concentration were positively associated with voriconazole concentration. The positive association between age and voriconazole concentrations has been well described. Elderly patients, who often have decreased liver function, are known to be at risk of higher voriconazole exposure [16, 17]. Similarly, an increase in CRP, as a marker of inflammation, has also previously been associated with increased voriconazole exposure, supposedly because of downregulation of CYP enzymes [18].

Despite identifying a negative association of 7‐day metamizole cumulative dose with voriconazole exposure, voriconazole Cmin was not found to be numerically different between the control and the concomitant group. However, some imbalances between the groups, notably in CRP, could have masked the effect of metamizole. The strong inflammatory response, as indicated by elevated CRP levels, may have counteracted the CYP‐inductive effects of metamizole. This is supported by the significant effect of both CRP and metamizole on voriconazole concentrations in the GEE model. For drugs with complex pharmacokinetics, such as voriconazole, sufficiently large cohorts are recommended to enable multivariable analyses. These analyses uncover associations that, in simple comparisons, may be obscured by multiple confounders. Likewise, because of retrospective design, data collection was limited, leading to potential confounding factors that could not be controlled for and may have impacted the findings.

The multivariable analysis of isavuconazole showed no significant association between the measured concentrations and the 7‐day cumulative metamizole dose but revealed a statistically significant association with sex, body weight, and ICU admission. Even though isavuconazole is extensively metabolized by CYP3A4, concomitant therapy with metamizole had no influence on isavuconazole concentrations. We hypothesize that this might be the consequence of isavuconazole's long half‐life (120 h) [9] It takes approximately 20–25 days to reach a steady state concentration of isavuconazole following dose adjustments. Thus, observation over a longer period would be needed to correctly understand the impact of pharmacokinetic changes. General patient characteristics, i.e., sex [19], body weight [9], and ICU admission [9, 10, 20], will have a more meaningful effect on the early pharmacokinetic profile of isavuconazole. Furthermore, metamizole was shown to induce CYP enzymes in a dose‐dependent manner [7], but the cumulative 7‐day metamizole dose was lowest in the isavuconazole group. Hence, these limitations could explain the lack of association found in our analysis.

The univariate analyses revealed a significant effect of the cumulative 7‐day metamizole dose and the daily posaconazole dose on the posaconazole concentrations. This analysis was severely limited by the sample size; nonetheless, the output indicated a negative association of metamizole with posaconazole concentration. Posaconazole is primarily metabolized by UGT [9, 21], however, some minor metabolization by CYP3A4 has been described [22]. This could suggest that CYP induction could contribute to the observed reduction in Cmin. However, the primary enzymes involved in the metabolism of posaconazole are the UGTs. The expression of these enzymes is in part regulated by CAR [23]. Thus, considering the role of CAR in metamizole‐related CYP induction, metamizole and/or its metabolites could also induce UGT expression.

Treatment and prevention of invasive fungal infections rely on adequate exposure to the antifungal triazole. TDM of triazoles is generally recommended to ensure efficacy and safety, or to account for high pharmacokinetic variability [10]. Our results suggest that concomitant use of metamizole can introduce additional intra‐ and interpatient variability in exposure of voriconazole and posaconazole. An effect of isavuconazole could not be detected in our study but might be masked by its long half‐life and the lower cumulative dose of metamizole used in these patients. Subtherapeutic exposure following metamizole‐mediated CYP‐induction may lead to treatment failure and/or development of azole resistance [10]. In vitro research in hepatocytes could confirm and elucidate the mechanism behind these findings. Additionally, prospective observational trials would provide much‐needed pharmacokinetic data to describe the in vivo extent of the interaction. In the meantime, we advise increased caution and a TDM‐based dosing approach, or, when triazole TDM is unavailable, to consider alternative analgesic treatment options.

Our study has several limitations. First, the retrospective design limited the data collection and interpretation. For example, we did not collect data on albumin levels, which are known to influence the pharmacokinetics of isavuconazole [20]. Second, the limited window of metamizole use in this population potentially prevented us from capturing a potential effect of metamizole on isavuconazole metabolism. Third, we could not account for any potentially relevant CYP‐enzyme polymorphisms. For voriconazole, the CYP2C19 genotype is known to significantly influence metabolism. Moreover, CYP3A4/5 polymorphisms could also be relevant, such as the CYP3A4*22 or CYP3A5*3 allele [24]. Fourth, we did not correct for multiplicity. Considering the exploratory nature of the study, the increased risk of false positives was considered acceptable in order to minimize the likelihood of overlooking potentially meaningful associations. Finally, GEE linear models were used to perform the multivariable and univariate analyses. This type of equation, however, requires complete data, resulting in some datapoints being lost in the statistical analysis because of missing data.

## 5 Conclusion

Use of metamizole in the 7 days prior to routine TDM sampling was associated with lower routine concentrations of posaconazole and voriconazole. Metamizole increases elimination of the triazoles, presumably through induction of CYP and UGT, in a dose‐dependent manner. Given the increased pharmacokinetic variability associated with concomitant metamizole use, concomitant use should be avoided in the absence of therapeutic drug monitoring.

## Author Contributions

**Bart Morlion:** writing – review and editing. **Kristina Nadrah:** methodology, investigation, writing – review and editing, data curation, conceptualization. **Ine De Booser:** investigation, methodology, writing – review and editing, writing – original draft, data curation, formal analysis. **Matej Somrak:** investigation, writing – review and editing. **Isabel Spriet:** writing – review and editing, conceptualization, supervision. **Katja Kalan:** writing – review and editing, investigation. **Matthias Gijsen:** writing – review and editing. **Katrien Lagrou:** writing – review and editing. **Steffen Rex:** writing – review and editing. **Saba Battelino:** writing – review and editing, investigation. **Johan Maertens:** writing – review and editing. **Aleš Matos:** writing – review and editing, investigation.

## Funding

The authors have nothing to report.

## Conflicts of Interest

The authors have no conflicts of interest to declare that are relevant to the content of this article. The authors report the following interests. Grants or contracts from entities unrelated to this manuscript were received by Katrien Lagrou from TECOmedical, and by JH from F2G, Gilead Sciences, and Mundipharma. Consulting fees were received by Bart Morlion from Opella, Grünenthal, Viatris, Haleon, and GSK; by Steffen Rex from Viatris; by Katrien Lagrou from Mundipharma; and by JH from F2G, Takeda, Mundipharma, and Basilea. Payments or honoraria for lectures, presentations, speakers bureaus, manuscript writing, or educational events were received by Bart Morlion from Viatris, Haleon, GSK, and Novartis; by Steffen Rex from Aguettant; by Katrien Lagrou from Gilead, Mundipharma, and FUJIFILM Waki Chemical Europe GmbH; and by JH from Mundipharma, Takeda, and Basilea. Payment for expert testimony was received by Bart Morlion from Sanofi. Support for attending meetings or travel was received by Kristina Nadrah from IATDMCT; by Steffen Rex from Aguettant and Viatris; by Katrien Lagrou from Gilead, Pfizer, and AstraZeneca; and by Isabel Spriet from Gilead. Regarding participation on Data Safety Monitoring Boards or Advisory Boards, Katrien Lagrou served on an advisory board for Pfizer. Finally, under other financial or non‐financial interests, Kristina Nadrah reports membership in the CHMP, ETF, and HCPWP at the EMA.

## Supporting information


**Table S1:** Dosing characteristics for each azole by target attainment.

## Data Availability

The data that support the findings of this study are available on request from the corresponding author. The data are not publicly available due to privacy or ethical restrictions.
