# Supplementary material for: Impact of Concomitant Metamizole Treatment on the Exposure of Mould‐Active Triazoles
Source: Mycoses. 2026 May 14;69:e70173. doi: 10.1111/myc.70173 (PMC13175134; doi:10.1111/myc.70173)
Supplement: Supplementary file 1 — Table S1: Dosing characteristics for each azole by target attainment. [file MYC-69-e70173-s001.docx]

**Impact of concomitant metamizole treatment on the exposure of mold-active triazoles
Authors:**

Dorian Vanneste^1^, Ine De Booser^1*^, Kristina Nadrah^2^, Matej Somrak^3^, Matthias Gijsen^1,4^, Aleš Matos^5^, Katja Kalan^6^, Bart Morlion^7,8^, Steffen Rex^7,8^, Saba Battelino^9,10^, Katrien Lagrou^11,12^, Johan Maertens^13,14^, Isabel Spriet^1,4^

*Shared first authors

Affiliations:

1. Department of Pharmaceutical and Pharmacological Sciences, Clinical Pharmacology and Pharmacotherapy, KU Leuven, Leuven, Belgium. [dorian.vanneste@uzleuven.be](mailto:dorian.vanneste@uzleuven.be).
2. Department of Infectious Diseases, University Medical Center, Ljubljana, Slovenia
3. Institute of Clinical Chemistry and Biochemistry, University Medical Center Ljubljana, Slovenia
4. Pharmacy department, University Hospitals Leuven, Leuven Belgium
5. Grošelj Matos otorinolaringologija, Ljubljana, Slovenia
6. Pharmacy department, University Medical Centre, Ljubljana, Slovenia
7. Department of Anaesthesiology, UZ Leuven, Leuven, Belgium
8. Department of Cardiovascular Sciences, KU Leuven, Leuven, Belgium
9. Department of Otorhinolaryngology and cervicofacial surgery, University Medical Center Ljubljana, Slovenia
10. University of Ljubljana, Faculty of Medicine, Department of Otorhinolaryngology, Ljubljana, Slovenia
11. Department of Microbiology, Immunology and Transplantation, KU Leuven, Leuven, Belgium.
12. Department of Laboratory Medicine and National Reference Centre for Mycosis, University Hospitals Leuven, Leuven, Belgium.
13. Haematology Unit, UZ Leuven, Leuven, Belgium
14. Department of Microbiology, Immunology and Transplantation, KU Leuven, Leuven, Belgium

Key words: Mold-active triazoles, metamizole, Drug interactions

Supplementary Table 1: Dosing characteristics for each azole by target attainment.

| **Control group** | **Voriconazole** | | | **Isavuconazole** | | | | **Posaconazole** | |
| --- | --- | --- | --- | --- | --- | --- | --- | --- | --- |
|  | **Sub-therapeutic (N = 38)** | **Therapeutic (N = 59)** | **Supra-therapeutic (N = 14)** | | **Sub-therapeutic (N = 17)** | **Therapeutic (N = 52)** | **Supra-therapeutic (N = 1)** | **Sub-therapeutic (N = 2)** | **Therapeutic (N = 9)** |
| **24h triazole dose (mg)** | 600  [400-782] | 640  [454-800] | 710  [450-1000] | | 200  [200-200] | 200  [200-300] | 150 | 400  [400-400] | 300  [300-400] |
| **Weight-adjusted triazole dose (mg/kg)** | 7.87  [6.14-10.8] | 8.42  [7.24-10.3] | 9.21  [7.77-10.7] | | 2.96  [2.30-4.26] | 3.70  [3.23-5.41] | 1.95 | 5.71  [5.71-5.71] | 4.10  [3.94-5.26] |
| **Concomitant group** | **Voriconazole** | | | **Isavuconazole** | | | | **Posaconazole** | |
|  | **Sub-therapeutic  (N = 42)** | **Therapeutic  (N = 61)** | **Supra-therapeutic  (N = 25)** | | **Sub-therapeutic  (N = 19)** | **Therapeutic  (N = 29)** | **Supra-therapeutic  (N = 5)** | **Sub-therapeutic  (N = 7)** | **Therapeutic  (N = 12)** |
| **24h triazole dose (mg)** | 600   [400-800] | 600  [400-720] | 520   [460-720] | | 200   [200-200] | 200   [200-250] | 200   [200-200] | 300   [300-300] | 300   [300-300] |
| **Weight-adjusted triazole dose (mg/kg)** | 7.73   [5.66-10.3] | 7.69   [6.17-8.16] | 7.99  [6.67-8.71] | | 2.60  [2.24-3.81] | 3.24  [2.58-4.34] | 2.89  [2.62-3.25] | 4.10  [3.35-4.29] | 4.09  [3.77-4.69] |
| **7-day cumulative metamizole dose (mg)** | 9500   [3630-18400] | 2000   [1000-4000] | 1500  [1000-5000] | | 1000   [750-3000] | 1500  [500-3500] | 3000  [2500-3500] | 7000  [3250-8250] | 1000  [500-1650] |
| Values are presented as median [Q1-Q3]. Target attainment | | | | | | | | | |
